# Supplementary material for: Epithelial sensing of vitamin A shapes intestinal antimicrobial defense
Source: bioRxiv. 2026 Mar 10:2026.03.08.710399. Preprint. [Version 1] doi: 10.64898/2026.03.08.710399 (PMC13060795; doi:10.64898/2026.03.08.710399)
Supplement: Supplement 1 [file NIHPP2026.03.08.710399v1-supplement-1.pdf]

bioRxiv preprint doi: https://doi.org/10.64898/2026.03.08.710399; this version posted March 10, 2026. The copyright holder for this preprint (which was not certified by peer review) is the author/funder, who has granted bioRxiv a license to display the preprint in perpetuity. It is made available under aCC-BY 4.0 International license.

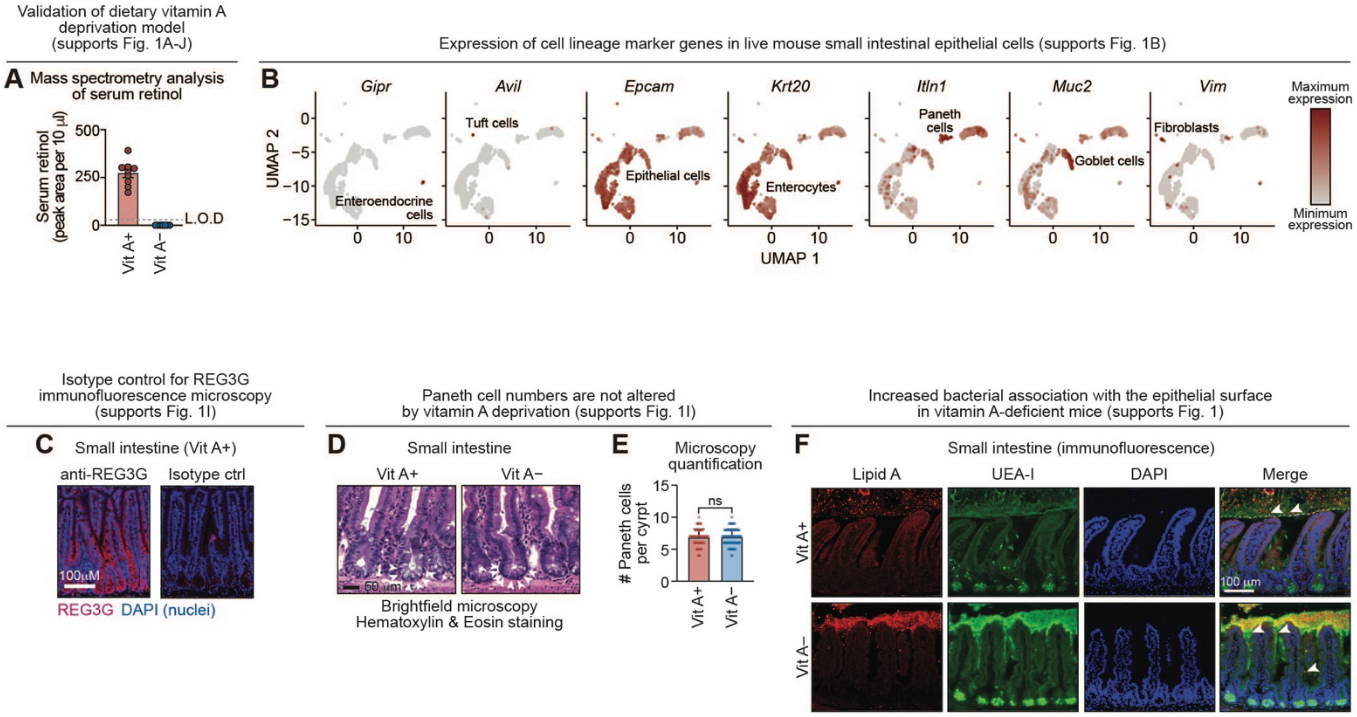

Figure S1. Characterization of the vitamin A deprivation model and expression of epithelial cell marker genes as determined by scRNAseq (supports Figure 1).

(A) Mass spectrometry measurement of retinol from serum of mice fed provided a Vit A+ (n=9) or Vit A– (n=14) diet as described in Fig. 1A. Each dot represents one mouse, and the limit of detection (L.O.D.) is indicated.

(B) Expression of marker genes among small intestinal cell populations as determined by scRNA-seq.

(C) Immunofluorescence microscopy of REG3G in small intestine sections from Vit A+ mice, with comparison to an isotype control antibody. Sections were stained for REG3G (red) and counterstained with DAPI (blue) to detect nuclei. Scale bar, 100 µm.

(D) Representative images of small intestinal crypts of mice fed a Vit A+ or Vit A– diet. Paneth cells were identified by their distinctive morphology and the presence of dense secretory granules (white arrowheads). Scale bar, 50 µm.

(E) Enumeration of Paneth cells identified in crypts from Vit A+ (60 crypts counted across 5 mice) and Vit A– mice (94 crypts counted across four mice).

(F) Immunofluorescence microscopy of lipid A (bacteria) and UEA-I (mucus) in small intestine sections from Vit A+ mice and Vit A- mice. The tissue was counterstained with DAPI to detect nuclei. The mucus barrier is outlined with a white dotted line and examples of positive lipid A staining are indicated by white arrow heads.

Vit A, vitamin A; L.O.D., limit of detection; REG3G, regenerating islet-derived protein 3γ; Ctrl, control; UEA-I, Ulex Europaeus Agglutinin I. Means ± SEM are plotted; ns, not significant by Mann-Whitney test.

bioRxiv preprint doi: https://doi.org/10.64898/2026.03.08.710399; this version posted March 10, 2026. The copyright holder for this preprint (which was not certified by peer review) is the author/funder, who has granted bioRxiv a license to display the preprint in perpetuity. It is made available under aCC-BY 4.0 International license.

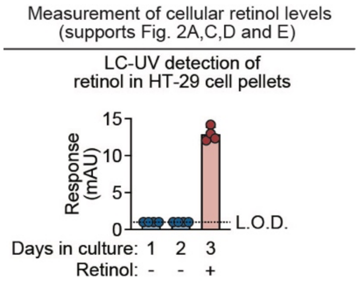

Figure S2. Characterization of retinol levels in HT-29 cells (supports Figure 2).
Liquid chromatography–UV spectroscopy analysis of cell pellets from HT-29 cells grown in DMEM with 10% charcoal stripped fetal bovine serum for three consecutive days. On day 3, 1 µM retinol was added to the culture medium and the cells were incubated for an additional 12 hours before analysis. Each data point represents one experimental replicate (n=4 per group).

LC–UV, liquid chromatography ultraviolet spectroscopy; L.O.D., limit of detection; mAU (milli absorbance units). Means are plotted.

bioRxiv preprint doi: https://doi.org/10.64898/2026.03.08.710399; this version posted March 10, 2026. The copyright holder for this preprint (which was not certified by peer review) is the author/funder, who has granted bioRxiv a license to display the preprint in perpetuity. It is made available under aCC-BY 4.0 International license.

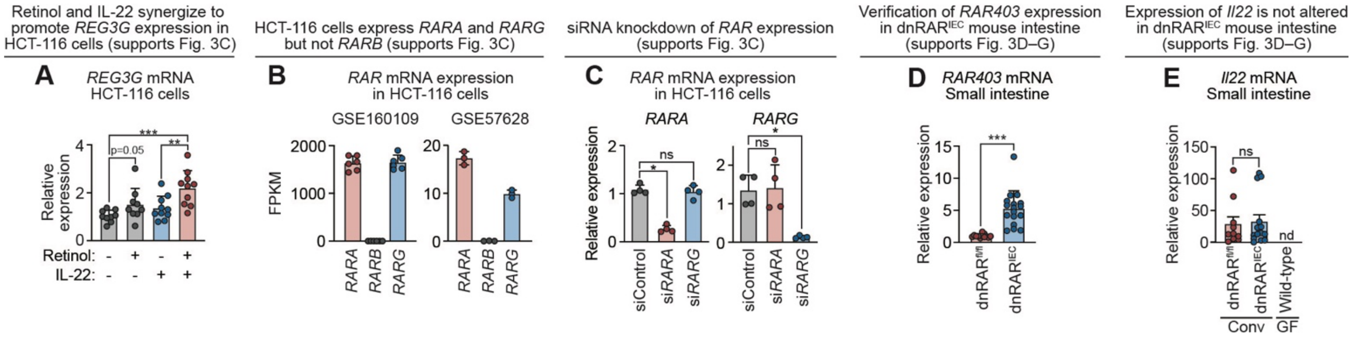

Figure S3. Characterization of *REG3G* and *RAR* expression in HCT-116 cells and validation of a genetic mouse model of RAR deficiency (supports Figure 3).

(A) qPCR analysis of *REG3G* transcripts in HCT-116 cells treated with or without retinol and/or IL-22. Each data point represents one experimental replicate (n=9 or 10 replicates per group).

(B) FPKM values of all three RAR gene paralogs (*RARA*, *RARB*, *RARG*) in HCT-116 cells. Data are from two independent and previously published RNA-sequencing studies (GSE160109 and GSE57628).[57] Each data point represents one experimental replicate (n=6 for GSE160109 and n=3 for GSE57628).

(C) qPCR of *RARA* and *RARG* expression 48 hours after siRNA treatment. Each data point represents one experimental replicate (n=4 per group).

(D) qPCR of *RAR403* transcripts in small intestines of conventional dnRAR$^{fl/fl}$ (n=12) and dnRAR^{IEC} (n=17) littermates (from five litters). Each data point represents one mouse.

(E) qPCR of Il22 transcripts in small intestines of conventional dnRAR$^{fl/fl}$ (n=10) and dnRAR^{IEC} (n=14) littermates (from five litters) and germ-free wild-type mice (n=8). Each data point represents one mouse.

REG3G, regenerating islet-derived protein 3γ; RAR, retinoic acid receptor; siRNA, small interfering RNA; dnRAR, dominant negative retinoic acid receptor; Conv, conventional; GF, germ-free; nd, not detectable. All qPCR measurements were performed in at least duplicate. Each data point represents one tissue culture well or one mouse where applicable. Means ± SEM are plotted; *p < 0.05; ***p<0.001; ns, not significant by Mann-Whitney test.

bioRxiv preprint doi: https://doi.org/10.64898/2026.03.08.710399; this version posted March 10, 2026. The copyright holder for this preprint (which was not certified by peer review) is the author/funder, who has granted bioRxiv a license to display the preprint in perpetuity. It is made available under aCC-BY 4.0 International license.

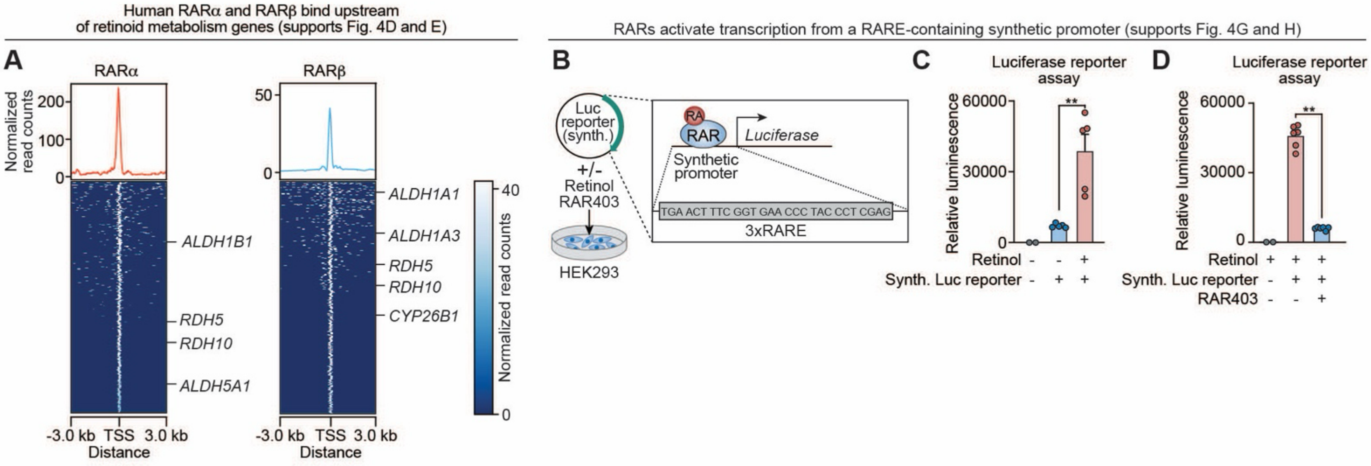

Figure S4. Controls for binding of RARα and RARβ in the human genome and characterization of transcription reporter assays (supports Figure 4).

(A) ChIP sequencing heatmap peaks centered around transcription start sites (TSS) generated via Deeptools[58]. Rows corresponding to retinoid metabolism genes are labeled.

(B) Transcription reporter assay with a synthetic RARE-containing promoter. HEK293 cells were transfected with a pGL2 plasmid containing a synthetic promoter sequence containing eight canonical RAREs positioned directly upstream of the firefly luciferase gene (synth. Luc reporter). The cells were treated with 1 μM retinol and/or co-transfected with a plasmid that expresses RAR403, which impairs RAR activity (see Fig. 3D).

(C) Luciferase reporter assay in the presence or absence of 1 μM retinol. HEK293 cells were transfected with the synthetic promoter–Luciferase reporter plasmid to establish that luminescence is dependent on the presence of the plasmid. Luciferase luminescence was measured in duplicate and each data point represents the average of two readings from one tissue culture well. (n=5 experimental replicates per group).

(D) HEK293 cells were transfected with the synthetic promoter–Luciferase reporter plasmid in the presence of 1 μM retinol and with and without RAR403. Luminescence was measured in duplicate and each data point represents the average readings from one tissue culture well. (n=6 experimental replicates per group).

RAR, retinoic acid receptor; TSS, transcription start site; Luc, luciferase; RA, retinoic acid; RARE, retinoic acid response element; Synth, synthetic. All luciferase measurements were performed in at least duplicate. Each bar graph data point represents one tissue culture well where applicable. Means ± SEM are plotted; $**p < 0.01$ by Mann-Whitney test.

bioRxiv preprint doi: https://doi.org/10.64898/2026.03.08.710399; this version posted March 10, 2026. The copyright holder for this preprint (which was not certified by peer review) is the author/funder, who has granted bioRxiv a license to display the preprint in perpetuity. It is made available under aCC-BY 4.0 International license.

Supplementary tables

Table S1: Oligonucleotides and primers

Gene target	Species	Sequence	Distributor and/or Citation
Reg3g	Mouse	Mm00441127_m1	Thermo Scientific #4351370
Reg3b	Mouse	Mm00440616_g1	Thermo Scientific #4448892
REG3G	Human	Hs01595406_g1	Thermo Scientific #4331182
RARE - 4195bp *Reg3g* promoter	Mouse	Forward: GCACGATGAACTTTCCCCAA<br>Reverse: TCACCTTGCAGGTCCATGAG	Integrated DNA Technologies Custom DNA Oligos
Off target *Vstm2a* promoter	Mouse	Forward: ACGCTGCCATTCTGATGACT<br>Reverse: ATTACGTAGGCCCTGGGGAT	Integrated DNA Technologies Custom DNA Oligos
Off target *Septin2* promoter	Mouse	Forward: CTCAAACACCAAGCCACGGT<br>Reverse: GCAGTGCAGATACACTTG	Integrated DNA Technologies Custom DNA Oligos
RARE - 143bp *REG3G* promoter	Human	Forward: TACCAGGCTCACAAGACTGC<br>Reverse: GACTCTTCCTGGCAAAGGCT	Integrated DNA Technologies Custom DNA Oligos
Off target *LINC01643* promoter	Human	Forward: TAGGCCTGTGTGTGGCAAAA<br>Reverse: TGGCTGTCTTCCCCGCTTAG	Integrated DNA Technologies Custom DNA Oligos
Off target *SEPTIN2* promoter	Human	Forward: CTCAAGGCTAGCATCCCGTC<br>Reverse: TCCCCAGAATTTTCAAGGATGC	Integrated DNA Technologies Custom DNA Oligos
Defa22	Mouse	Mm04206099_gH	Thermo Scientific #4331182
RARE - 1010bp *DEFA6* promoter	Human	Forward: GCCCACAATCCCCCTCTGAA<br>Reverse: AAGTGGAGATTTCCTGGAGCA	Integrated DNA Technologies Custom DNA Oligos
RARA	Human	Hs00940446_m1	Thermo Scientific #4331182
RARG	Human	Hs01559230_m1	Thermo Scientific #4331182

bioRxiv preprint doi: https://doi.org/10.64898/2026.03.08.710399; this version posted March 10, 2026. The copyright holder for this preprint (which was not certified by peer review) is the author/funder, who has granted bioRxiv a license to display the preprint in perpetuity. It is made available under aCC-BY 4.0 International license.

RAR403	Transgene	Forward: TGCTTGGCGAACTCCACAGTCTTA<br>Reverse: GCGCTCTGACCACTCTCCAGC	Rajaii et al., 2008[35]<br>Integrated DNA Technologies Custom DNA Oligos
Il22	Mouse	Mm01226722_g1	Thermo Scientific #4331182
Rn18s	Mouse	Mm03928990_g1	Thermo Scientific #4331182
18S	Human	Hs03003631_g1	Thermo Scientific #4331182
SFB DNA	Bacteria	Forward: GACGCTGAGGCATGAGAGCAT<br>Reverse: GACGGCACGGATTGTTATTCA	Brooks et al., 2021[9]<br>Integrated DNA Technologies Custom DNA Oligos
Eubacteria DNA	Bacteria	Forward: ACTCCTACGGGAGGCAGCAG<br>Reverse: ATTACCGCGGCTGCTGG	Brooks et al., 2021[9]<br>Integrated DNA Technologies Custom DNA Oligos

Table S2: LC-UV gradient program for retinol detection

Time (min)	Mobile A (%)	Mobile B (%)
0.0	100	0
2.5	0	100
8.5	0	100
8.6	100	0
10.0	100	0
